# Modeling and Risk Analysis of Chemical Terrorist Attacks: A Bayesian Network Method

**DOI:** 10.3390/ijerph17062051

**Published:** 2020-03-19

**Authors:** Rongchen Zhu, Xiaofeng Hu, Xin Li, Han Ye, Nan Jia

**Affiliations:** School of Information Technology and Network Security, People’s Public Security University of China, Beijing 102628, China; zhurongchen@stu.ppsuc.edu.cn (R.Z.); lixin@ppsuc.edu.cn (X.L.); Jianan121@ppsuc.edu.cn (N.J.)

**Keywords:** chemical terrorist attack, Bayesian network, risk analysis

## Abstract

The chemical terrorist attack is an unconventional form of terrorism with vast scope of influence, strong concealment, high technical means and severe consequences. Chemical terrorism risk refers to the uncertainty of the effects of terrorist organisations using toxic industrial chemicals/drugs and classic chemical weapons to attack the population. There are multiple risk factors infecting chemical terrorism risk, such as the threat degree of terrorist organisations, attraction of targets, city emergency response capabilities, and police defense capabilities. We have constructed a Bayesian network of chemical terrorist attacks to conduct risk analysis. The scenario analysis and sensitivity analysis are applied to validate the model and analyse the impact of the vital factor on the risk of chemical terrorist attacks. The results show that the model can be used for simulation and risk analysis of chemical terrorist attacks. In terms of controlling the risk of chemical terrorist attack, patrol and surveillance are less critical than security checks and police investigations. Security check is the most effective approach to decrease the probability of successful attacks. Different terrorist organisations have different degrees of threat, but the impacts of which are limited to the success of the attack. Weapon types and doses are sensitive to casualties, but it is the level of emergency response capabilities that dominates the changes in casualties. Due to the limited number of defensive resources, to get the best consequence, the priority of the deployment of defensive sources should be firstly given to governmental buildings, followed by commercial areas. These findings may provide the theoretical basis and method support for the combat of the public security department and the safety prevention decision of the risk management department.

## 1. Introduction

In recent years, terrorist attacks around the world have caused not only significant losses of life and property, but also a series of public problems such as public panic and social unrest. Chemical terrorist attacks apply to different terrorist purposes, which have been more frequently used because of the characteristics of easy availability of materials, significant effects, convenient implementation and difficult rescue [1]. According to the statistics published by the US Department of Homeland Security on “Global Chemical Attacks from 1950 to 2005,” there have been 423 nuclear, biological, and chemical terrorist incidents worldwide in 55 years, of which 239 were chemical terrorist attacks, accounting for 56% [2]. On average, each chemical terrorist incident resulted in 51 injuries and 7 deaths [3]. Furthermore, chemical terrorist attacks have diversified destructive capacity and pose a significant threat to human life, living environment, society, and psychology [4,5], such as the Tokyo subway sarin gas attack [6]. 

The above facts show that the consequences of chemical weapon attacks are often unbearable for the state, government, society, and the masses. Both industry and academic risk analysis groups have invested heavily in the development of tools and methods, hoping to mitigate the risks of terrorism and other threats to the country [7]. Since chemical weapon attack is not conventional, the barriers need to be moved forward. And the prevention initiative should be strengthened through monitoring and prediction to propose effective and proactive prevention strategies. Moreover, the response strategy and comprehensive plans should be developed and implemented to remedy the identified deficiencies as well as reducing loss after the attack [8]. 

Many characteristics of chemical terrorist attacks also determine the particularity of its risk evolution law, which needs to be studied intensively. First of all, chemical terrorist attacks are much more likely to happen in densely populated urban areas than remote areas with few people. The time and place of the incident largely depend on the subjective decisions of the terrorist attackers. The distribution and operation of high-value attack targets and defense forces in cities will also affect the decision to a certain extent. All these ultimately affect the risk distribution and evolution of chemical terrorist attacks. Secondly, as chemical substances can be released by various methods like volatilisation, water-solubility, spraying, and explosion dispersion [1], the diffusibility and spread of chemical substances provide favorable conditions for aggravating the consequences. Without any doubt, chemical terrorist attacks also have weaknesses. The manufacturing, storage, transportation, and release of chemical weapons require high technical capabilities and usually require the participation of multiple people. The chain of terrorist attacks is relatively long, which makes it easier to leave clues. Capturing these clues requires the support of multi-source data. Therefore, it is urgent to carry on risk analysis of chemical terrorist attacks in order to propose effective prevention strategies.

## 2. Related Work

Chemical terrorist attack is a complex process. Accurately identifying the risks can provide a solid basis for subsequent risk analysis. According to the “Risk management—Principles and guidelines” [9], the organisation should determine the source of risk, the scope of impact, events (including environmental changes) and their causes and potential consequences. Many previous studies [10,11,12,13] focused on different parts of chemical terrorist attacks. Evison et al. studied the definition of chemical weapons, methods of dissemination, the characteristics of different types of chemical weapons, and put forward preventive recommendations and emergency measures [1]. Szinicz reviewed the history of chemical and biological warfare agents [14]. Laurent et al. introduced the management of victims of urban chemical attack in French [15]. Bennett analysed the preparedness of hospitals for managing victims affected by chemical or biological weapons and concluded that hospitals continue need to require substantial resources at different levels to be “truly” prepared [16]. Greenberg et al. studied the preparedness of emergency departments in the greater Philadelphia area to evaluate and treat victims of a terrorist biological or chemical agent release [8]. Reniers analysed the possible terrorist attacks on the chemical industry park and proposed intelligently planning protection measures to prevent the domino effect [17]. 

The above researches not only provide a basis for the characteristics and spread law of chemical weapons, but also improve emergency decision-making and prevention strategies of chemical terrorist attack to a certain extent. However, it can be seen that many studies have often conducted in-depth analysis of risk factors in only one aspect. But few of them have considered all factors comprehensively. Therefore, our method performs a detailed risk identification. In this paper, risk factors for chemical terrorist attacks are divided into the following categories. Sources of risk: Characteristics of terrorist organisations and chemical weapons, Scope of influence: information on the targets and the operating environment of the city. Environmental changes: climate conditions, emergency response forces, and police prevention forces. Accident consequences: the probability of successful attacks and casualties.

After identifying the risk factors of chemical terrorist attacks, we need to find an effective risk analysis method. Many scholars have studied the modeling and analysis methods of terrorism [18,19,20,21,22], such as decision making system [23] and game theory [24]. Especially, Regens et al. assessed the differences in law enforcement officers’ response to terrorism-centric cognitive judgments and decisions in their daily policing. The authors used Bayesian network for modeling and Monte Carlo simulations, which ultimately led to the interaction between jurisdiction, training, experience, and familiarity with terrorism [25]. White et al. explored the patterns of terrorist activity in three countries (Indonesia, the Philippines, and Thailand) from 2000 to 2010. The authors used a self-excited point process model to create interpretable and reproducible metrics for terrorism from risk, resilience, and volatility. The article concluded that the risk, volatility and resilience indicators of these three countries have significant differences over time [26]. Willis et al. described a method of using probabilistic risk modeling in break-even benefit-cost analyses of terrorism security regulations [27]. Ezell et al. explored many existing and potential terrorist risk analysis methods, particularly concerning probability and decision analysis methods [7]. However, these methods have limitations when modeling terrorist risk: (1) Unable to deal with the uncertainties related to the complex interaction factors of terrorist organisations and other factors. (2) Unable to answer “hypothetical” questions, for example: Which measures, such as security checks, surveillance, can reduce the attack risk of specific terrorist organisations?

We use Bayesian Network (BN) to take multi-source information into consideration and study the probability of the occurrence and consequences of chemical terrorist attacks. Bayesian network (commonly referred to as BN) is a probabilistic graph model which consists of nodes and edges. Nodes represent variables and edges represent the relationships between variables. The relationships between nodes are described in the form of directed edges with conditional probability distributions [28]. The probability distribution can be obtained through expert experience or sample learning [29]. BN has a solid theoretical foundation and is convenient for processing incomplete data. It can deal with uncertainty and update the probability of events [30]. A Bayesian network generally consists of several parts: (a) variables and directed links between the variables; (b) Each variable has a finite set of mutually exclusive states. (c) an assigned conditional probability for each variable with ‘parents.’ BN is able to reason under uncertainty and update its predictions based on new observations [31]. Users can enter the prior information of one or more variables in BN to update the probability of other nodes. BN, as a common decision-making method, is also widely used in many fields [32,33,34,35]. Bayesian network was used to develop a quantitative method for assessing the vulnerability of chemical facilities to external attacks [36]. Landucci et al. evaluated the attack likelihood contribution to security risk and used a probabilistic risk analysis method to analyse chain events based on BN [37]. Based on the combination of probabilistic risk analysis and Bayesian network, Pate-Cornell and Guikema studied the impact of terrorist attackers’ decisions on emergency decision-makers, providing methodological support for assessing possible loss and formulating prevention strategies [38]. However, the data used in Pate-Cornell and Guikema’s research are all hypothetical which lack authenticity. [39] and [40] used the historical data in the Global Terrorism Database (GTD) [41] to evaluate the diversity, uncertainty, and ambiguity of terrorist attack assessment information, and proposed a terrorist attack risk assessment method and prevention strategy based on BN. Olama et al. proposed a BN based terrorist threat anticipatory model, which takes the physical, social, and economic aspects into consideration [42]. But he did not analyse the target’s defense capabilities and emergency response capabilities.

In a word, the researches on chemical terrorist attacks have had the foundation and accumulation in theories and methods, but there are still some deficiencies. The main contribution of this article is:

(1) Based on real case data and expert experience, a Bayesian network is constructed to model the risk of chemical terrorist attacks in depth.
(2) We model and analyse chemical terrorist attack risk from multiple sources, such as terrorist organisations, defensive forces, and urban environments to capture the dynamic evolution of risk and propose prevention strategies.

The rest of this article is structured as follows. The detail of Bayesian network construction will be introduced in Section 3. In Section 4, we apply the proposed model to Lianyungang city to verify the model and complete the discussion. Finally, we conclude and describe the limitations and scope of further research.

## 3. Bayesian Network Construction

The following subsections present the methodology of Bayesian network for chemical terrorist attacks. As we discussed above, the risk of chemical terrorist attacks is affected by many factors, so it is difficult to consider all factors at a time. To solve the problem of building complex Bayesian networks, Laskey et al. [43] proposed the idea of modular construction. And we construct Bayesian networks from the perspectives of terrorist organisations, weapon, target, weather, prevention, emergency response, and consequence respectively, then combine them into a complete Bayesian network.

We use multi-source data to build Bayesian network. (1) GTD terrorist attack data. The Global Terrorism Database (GTD) is an open database containing information on global, domestic, and international terrorist attacks from 1970 to 2018, and currently has more than 190,000 cases. Each case provided information on the date, location, type of chemical weapon, number of casualties, and terrorist organisations. (2) Wikipedia and Baidu Encyclopedia. We collect keywords and search in encyclopedias, such as chemical weapons: mustard gas, sarin gas; attacks: Japanese subway sarin gas, Matsumoto sarin incident; terrorist organisation: ISIS, Taliban; (3) News reports. We use crawlers to crawl reports and news about chemical terrorist attacks on news sites such as China Caixin.com (4) Literature and reports on official government websites. All data is saved in a relational database for easy retrieval. Other city information, defense force information, and emergency response information come from the actual situation of the case studied. Except for information on public security patrols, surveillance, investigations, and security checks, as well as emergency response situations in each specific area, other information is publicly available.

### 3.1. Steps to Construct Chemical Terrorist Attack Bayesian Network

**Figure 1:**
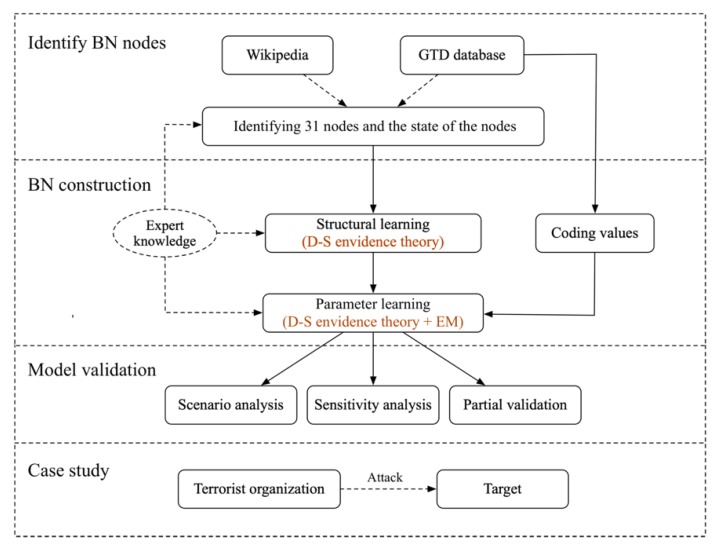
The process flow of framework.

Figure 1 shows the steps to construct BN: (1) Determine the BN variables and their state classification based on multi-source data and expert knowledge; (2) Determine structure and probability distribution of BN. In this paper, we firstly collect questionnaire results of four experts, and take advantage of D-S (Dempster-Shafer) evidence theory [44,45] to analyse the causal relationship between BN variables. Then we determine the conditional probabilities of all nodes. (3) We perform model validation with qualitative (scenario analysis) and quantitative (sensitivity analysis and partial validation) methods [46,47]. After finishing the above steps, the BN with probabilities can be used to conduct case studies.

### 3.2. States of the Parent and Child Nodes

In order to thoroughly analyse the risk of chemical terrorist attacks, it is necessary to understand all aspects of the threat elements. Through analysis of historical case in Global Terrorism Database (GTD) [41], a summary of terrorism news, some cases’ and terrorist organisations’ descriptions in Wikipedia, plus expert experience and knowledge contained in the literature, we propose 31 parent nodes (nodes 1-31) and 11 child nodes (nodes A-K). The BN nodes are shown in Table 1 and described in Appendix A. These nodes describe the attack in terms of attack subject, attack object, attack consequence, and objective conditions. However, it should be noted that in Hazard and Operability Analysis (HAZOP) and Safety Integrity Level Analysis (SIL), incident consequences are often quantified from property loss, human damage, and environmental impacts [48,49]. In a chemical weapon attack, the characteristics of chemical weapons, the dose of weapons, the method of delivery, and the mode of diffusion of chemical substances in the atmosphere have a significant impact on the environment. However, such information is lacking in the collected cases, and the focus of this article is on the overall modeling, so we do not consider environmental impact. More researches can be carried out based on the dirty bomb diffusion model [50,51,52]. Property loss includes the cost of treatment for the victims and personal property loss. However, the property loss of most attacks is difficult to quantify, we only consider the casualties as a consequence.

### 3.3. Determination of BN Structure

Two methods are used to build the network. The network structure between child nodes D, E, F, G, H, I, J, K is derived using expert experience, and the structure between other nodes was obtained from literature research and brainstorming of the research team. Four experts participate in the network building, and each of them has extensive hands-on experience in police information, chemical terrorist attacks, and risk assessment. We collected the professional judgments of four experts through a questionnaire survey. The experts were asked if there were influences in the pair of child nodes and assigned values. To reduce the subjectivity of expert opinions, we use D-S evidence theory to analyse the collected data. The D-S evidence theory defines a frame of discernment H and the Mass function. The Mass function needs to satisfy the following conditions:

(1) $$\{\begin{array}{l} m(\phi )=0\\ \sum_{A\subseteq \Theta }m(A)=1 \end{array}$$

where m(A) is the Mass function of event A, which is also the basic probability function of discernment Θ, the synthesis rule of Dempster-Shafer evidence is shown in Equation (2).

(2) $$m(A)=\{\begin{array}{l} \frac{1}{1-K}\sum_{A_{1}\cap A_{2}\cap \ldots \cap A_{N}}m_{1}\left(A_{1}\right)m_{2}\left(A_{2}\right)\ldots m_{N}\left(A_{N}\right),A\neq \phi \\ 0,A=\phi \end{array}$$

where, A1, A2, … AN represent different states of the event, m1, m2, …, mN are the basic probability functions of discernment Θ, and K represents the conflict degree among m1, m2, …, mN, which is calculated as follows:

(3) $$\begin{array}{ll} K & =\sum_{A_{1}\cap A_{2}\cap \ldots \cap A_{N}\neq \phi }m_{1}\left(A_{1}\right)m_{2}\left(A_{2}\right)\ldots m_{N}\left(A_{N}\right)\\ & =1-\sum_{A_{1}\cap A_{2}\cap \ldots \cap A_{N}=\phi }m_{1}\left(A_{1}\right)m_{2}\left(A_{2}\right)\ldots m_{N}\left(A_{N}\right) \end{array}$$

We use the “K. Casualties” node as an example to describe how we obtain the causal relationship between nodes through expert experience. m1(1,2), …, m4 (1, 2) represent the probability whether the node J and the node K are related by the four experts. The value of m1 (1, 2) is (0.9, 0.1), which means that the first expert thinks “J. Whether the attack is successful” has a great influence on the “K. Casualties”. The probability of a strong relationship between these two nodes is 0.9, while the probability of a weak relationship is 0.1. The four experts give their respective values. Then we calculate the weight of causality between two nodes based on equations (2) and (3). If the weight of the causal relationship between two nodes is larger than the threshold value (0.85), the relationship exists.

**Figure 2:**
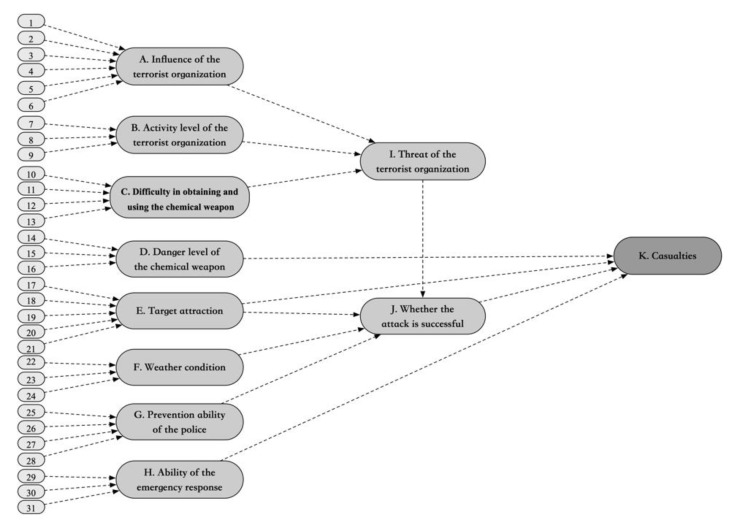
Bayesian network for representing chemical terrorist attack. The description of each node is shown in Table 1.

Based on the experience of four experts, the Bayesian network is shown in Figure 2. As a result, 11 child nodes and 31 independent parent nodes are connected through 42 causal relationships. 

### 3.4. Determination of Probability Distributions of BN Nodes

The Bayesian network probability acquisition methods mainly include the method of sample learning and the method relying on expert experience to build. The former applies to the case where there is sufficient sample data, and the latter applies to the case where the domain expert gives the node probability distribution. As we lack case data that include all nodes, we cannot perform sample learning on all nodes. So, we use GTD data and Wikipedia data to finish sample learning for the prior probabilities of parent nodes 1-16 and the conditional probability of child nodes A, B, C, D, E, I. The prior probability of the parent node 19-31 is assigned according to the actual situation of the case studied, and the conditional probability of the child nodes E, F, G, H, J, K come from expert experience.

#### 3.4.1. Learning Probability Distributions Based on Samples

In order to use sample learning, we need to perform data discretisation. We recruited five programmers to discrete 330 chemical terrorist attacks cases in the GTD database. All the programmers are undergraduates from the People’s Public Security University of China. After data screening and preprocessing, 287 cases were finally obtained. We use the EM algorithm [53] included in GeNIe [54] software to complete parameter learning. Since the data sets required for Bayesian network parameter learning usually have varying degrees of data loss, parameter learning becomes difficult. The EM algorithm is based on maximum likelihood to solve the parameter learning problem with hidden variables.

#### 3.4.2. Learning Probability Distributions Based on Expert Experience

In Table 2, we use D-S evidence theory to analyse the probability distribution of the “H. Ability of the emergency response” node as an example. In the second round of questionnaires, we collected the conditional probability of the nodes, then processed the results using the Dempster-Shafer evidence method. 

### 3.5. Model Validation

We validate the model from scenario analysis, sensitivity analysis, and partial validation.

#### 3.5.1. Expert Validation of Scenarios

In this process, different hypothetical scenarios are considered. The first is the worst state, which means that all parent nodes are set to the most threatening situation. For example, we set the number of terrorist organisations to the maximum and the technical background to the high. The second is the best state. The node state settings for the two extreme conditions are shown in Table 3.

In these two extreme conditions, the three states of casualties are estimated as (minor, medium, major) = (0.24, 0.16, 0.6) and (1, 0, 0), respectively. The result means that in the worst case, the probability of “casualties” = “major” is 0.6, and in the best condition, the probability is close to 0. The worst and best probability distribution of “J. Whether the attack is successful” is (0.86, 0.14) and (0, 1). Extreme condition tests show that the Bayesian network is normal and effective.

Then, we consider four hypothetical scenarios, not just two extreme conditions. Table 4 shows the status of node “J. Whether the attack is successful” and “K. Casualties” for the four scenarios. Due to word limitations, we have not considered all possible situation.

Scenario 1 represents the most common type. We set node 1–21 to the state with the most common initial probability value and ensure that there are no contradictions in all states. Then we observe the probability distribution of the child nodes. The probability of successful attack is 46%; the probability of failure is 54%, and the difference is 8%, which illustrates that attacks are more likely to fail. Among the casualties, 69% are minor, 27% are medium, and 4% are major, which indicates that there is a higher probability of fewer casualties in the most common chemical terrorist attack.

Scenario 2 sets the weapon type to neurotoxin based on scenario 1, because neurotoxin is the most severe type of chemical weapon. We find that minor casualties fell from 69% to 61%, while major increased from 4% to 23%, which means neurotoxins are more likely to cause high mean number of injuries and fatalities [3].

Scenario 3 observes the impacts of weather condition. We set “Weather condition” to “favourable” based on scenario 1. Then the probability of successful attacks increased from 46% to 51%. Then the state is set to “unfavourable”, the probability of successful attack drops from 46% to 30%, and the probability of unsuccessful attack increases from 54% to 70%. The result reflects that weather condition has some impacts in the success of the attack, which explains why many terrorists attack in weather conditions conducive to the spread of poisons. Nuclear material can spread faster and farther in favourable weather condition or environment and cause more severe consequences [52]. Similar characteristics appear in the diffusion of chemicals.

Scenarios 4 sets the highest police preventative capability based on scenario 1. This time the probability of successful attacks dropped from 46% to 24%, and the probability of unsuccessful attacks has increased from 54% to 76%, which also reflects that the police preventative ability has greatly affected the success of the attack.

All four scenarios represent different states of the Bayesian network. We consider different combinations of parameters to generate different scenarios and observe the rationality of the probability distribution in the incident result node to complete the model verification.

#### 3.5.2. Sensitivity Analysis

In this paper, the sensitivity analysis method is used to measure the influence of small changes in parameters in the Bayesian network on the target probability value or distribution.

We perform a sensitivity analysis on the “Casualties” node. From Table 5, we find that when changing the “Security check” from “No” to “Yes”, the probability of “Casualties” = “minor” changes the most, increasing from 66% to 90%. Similarly, if “Police Investigation” is changed from “No” to “Yes”, the probability of “Casualties” = “minor” increases from 71% to 88%. When the state of the “Surveillance” changes, the probability of “Casualties” = “minor” increases from 61% to 77%. We can see different police prevention capabilities have a different impact on the casualties. Security check has the most apparent effects on the reduction of casualties. The effects of the police investigation, patrol, and surveillance have gradually declined, which is consistent with the experience of experts, because security checks often consume more defense resources and the effect are apparent. Security checks and police investigations serve as a precautionary measure, while patrol plays a deterrent role. And surveillance with early warning function have limited the reduction of casualties. Table 5 and Table 6 shows the sensitivity of “Casualties” and “Whether the attack is successful” to patrol, security check, surveillance, police investigation is the same, which also accords with the three axioms of Section 3.5.3. For the state of “Casualties” = “major”, when the emergency response of the hospital, fire, and police changed from “delay” to “on time”, the probability dropped from 25% to 10%, and when “security check” changes from “No” to “Yes” at the same time, the probability value dropped from 10% to 5%, which shows that the combination of pre-event prevention and post-event response can more reduce casualties.

However, we should note that economic conditions are limited in many regions, so that many measures cannot directly go from 0 to 1. Therefore, we let the initial probability of the parent nodes change in the range of 10% or 20%, so that it’s more practical for measure evaluation. If the values of all parent nodes change only within 10% of the prior probability distribution, for “Casualties” = “minor”, “Security check”, “Patrol”, “Surveillance” are most sensitive to the state. The degrees are 3%, 2.2% and 2%. Similarly, we can compare inputs and outputs and find the node that have the most significant impact on casualties within a certain range of changes.

The results of the sensitivity analysis are consistent with expert judgment, which reflects the rationality of our model.

#### 3.5.3. Partial Validation

In this study, we use a partial verification method based on the three axioms to validate the model. The three axioms are proposed by Jones et al. [55] and used in Kabir’s work [47]:

(1) If the prior probability of the parent node slightly decreases/increases, the posterior probability of the corresponding child node should also change accordingly.
(2) The change in the probability distribution of the parent node should have a consistent impact on the child nodes.
(3) The total impact of the combination of probability changes from m attributes on the value should always be greater than from the set of m − n (n ∈ m).

For example, considering the “G. Prevention ability of the police”, when the prior probability of the state “Security check” = “Yes” is set from 40% to 45%, the probability of “G. Prevention ability of the police” = “High” increases from 57% to 60%, the probability of “J. Whether the attack is successful” = “No” increased from 68% to 70%. Based on this change, when the prior probability of “Wind direction” = “Upwind” increases from 15% to 25%, the probability of “F. Weather conditions” = “Unfavorable” increases from 24% to 32%, and the probability of “J. Whether the attack is successful” = “No” increases from 70% to 71 %. The process of adding each influencing node satisfies the axiom and provides partial verification of the model. We perform similar analysis on other nodes.

## 4. Case Study: Haizhou District, Lianyungang City

Lianyungang city is located in Jiangsu Province, China. Haizhou District is the political, economic, and cultural center of Lianyungang City. According to the 2016 census, 830,000 people live in Haizhou District.

In the past few decades, a large number of chemical terrorist attacks have occurred around the world, each with different characteristics and targets. For preliminary research, we selected three representative locations in Haizhou District as hypothetical targets: Suning Square, the municipal government, and Phoenix Mountain Park. Then we choose three types of terrorist organisations. Through the combination of different organisations and targets, a total of nine scenarios are observed. Since weather conditions change with specific time and scene, it is difficult to set a fixed value. We assume that terrorists will choose favorable weather conditions to launch attacks.

### 4.1. Target Information

Target A: Suning square, the most crowded shopping mall in Haizhou District. Suning square is the first choice for people to go shopping in downtown. During morning and evening rush hours, traffic jams may occur. In front of Suning Square, there is a fixed police station for patrolling and deterrence. For the convenience of people shopping, the mall does not have any security checks, but the roads and the mall have high-definition surveillance. The fire brigade of the railway station is responsible for the fire emergency response of the shopping mall. It is about 3.1 km away and takes about 11 min by car. The Lunan Police Station is 900 m away and takes 2 min by car. The emergency rescue hospital is 1.7 km away and takes ten minutes by car. Under normal circumstances, the emergency response of the police, hospitals, and fire are timely, but in the morning and evening peak traffic jams, the ability will decline. This type of target is prevalent in urban chemical terrorist attacks. The main characteristics of this type of target are high population density, large population flow, good traffic conditions, mainly located in commercial areas. Even this target has strong emergency capabilities, short response times, and fast actions, once the attack is successful, the consequences can be severe. For example, on March 20, 1995, a sarin gas attack on the Tokyo subway during the early rush hour killed 13 people, severely injured 50 people, and poisoned thousands [6].

Target B: Government of Lianyungang City. The government is located in the southeast of Haizhou District. There are many communities next to the government. The population is moderate, and the security check will be carried out from time to time. The government’s police prevention capacity is almost the strongest in Lianyungang. The fire brigade is 4 km away. The police emergency response belongs to the Cangwu police station, which is 300 m away and takes 1 min by car. The hospital is 5.9 km away and takes 17 min by car. Due to the long distance and traffic jams, the emergency response of the hospital may be delayed. Attacking such targets by terrorists is very common and has a strong deterrent effect. The main characteristics are: the target has a political meaning, a moderate population density and mobility, and good traffic conditions. Police have a strong ability to combat and prevent.

Target C: Phoenix Mountain Park, a park in the suburbs with few people and bad traffic. It can be seen that in addition to the emergency response capabilities of police, the capabilities of hospitals and firefighting are not good enough, too. At the same time, in such parks, there are no effective patrol, investigation, and security measures, so the police’s prevention ability is insufficient. With low attraction, such target is rarely attacked.

**Figure 3:**
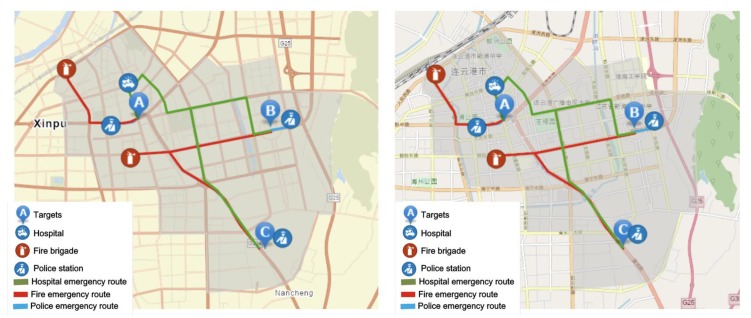
Hospital, fire brigade, police station and Target A, B, C of the City.

Table 7 shows the node status of each target, and the position information of the three targets is displayed in Arcgis in Figure 3.

### 4.2. Terrorist Organisation Information

We assume that there are three different types of terrorist organisations, and the node status is shown in Table 8.

Organisation 1: This type of organisation is characterised by: large scale, very active, medium cultural level and technical background. Most of the chemical weapons they have are acquired through occupation. An example is the infamous and cruel ISIS terrorist organisation in Syria. There were 41 ISIS chemical terrorist attacks shown in the GTD.

Organisation 2: This type of terrorist organisation has the following characteristics: the number of people is medium, the social relations and organisational components are medium, inactive, and it is difficult for public security agencies to detect and prevent in advance; such organisations have a low cultural level and no technical background. They obtained chemical weapons through theft and invasion. What we need to pay attention to is that many small and medium-sized cities have some chemical companies. Due to the loose corporate supervision, terrorist organisations can obtain raw materials from chemical plants and launch terrorist attacks. The Bhopal disaster in India is a real case. On the evening of December 2, 1984, pesticides from chemical plants were leaked in the Bhopal region of India. More than 500,000 people were exposed to methyl isocyanate [8] gas in the incident. Highly toxic substances enter the small town near the factory and its surroundings. The leak injured 558,125 people, including temporary local injuries to 38,478 people. The Indian government and local activists believe that negligent management and postponed maintenance have caused the disaster. Learning from this case, if terrorists use management loopholes to enter chemical plants easily, a similar disaster may happen.

Organisation 3: The main features are small scale, good at camouflage and hiding, high technical background, high cultural level, and having self-made chemical weapons. For example, Japan’s Aum Shinrikyo, the core members of the organisation have launched two appalling chemical terrorist attacks: Matsumoto Nagano sarin gas attack and Tokyo subway sarin gas attack.

### 4.3. Results

**Figure 4:**
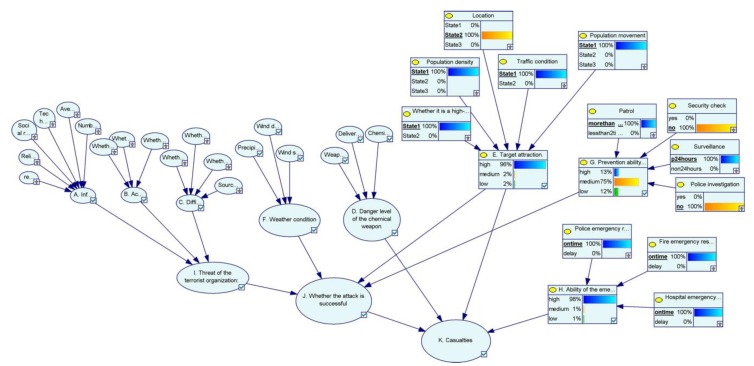
Node status of Target A in Bayesian network.

We set evidence for each of the nine scenarios (Table 9), and then, to observe and discuss the distribution of node states. In Figure 4, we show the node status of target A in the Bayesian network.

**Figure 5:**
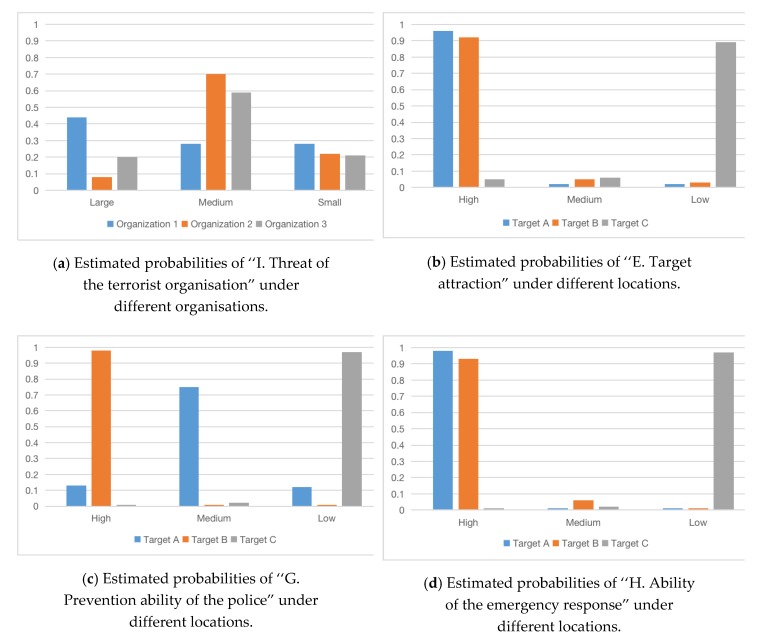
Estimated probabilities of nodes I, E, G, H under different organisations and targets.

From Figure 5a, we can see the probability of “I. Threat of the terrorist organisation.” Organisation 1: (Large, Medium, Small) is (0.44, 0.28, 0.28); Organisation 2: (Large, Medium, Small) is (0.08, 0.7, 0.22), Organisation 3: (Large, Medium, Small) is (0.2, 0.59, 0.21). Although Organisation 3 has a small number of people and is not very active, it is more threatening than Organisation 2 because of its high level of technology and the ability to independently manufacture chemical weapons. That means in chemical weapons attacks, terrorist organisations with self-manufacturing capabilities are more threatened. Organisation 1 has a large number of people, a large influence, and is extremely active. Although it does not have chemical weapon manufacturing technology, it can transport and launch weapons. We must cut off its acquisition of weapon manufacturing technology or high-level talents. Otherwise, the threat will be even greater.

**Figure 6:**
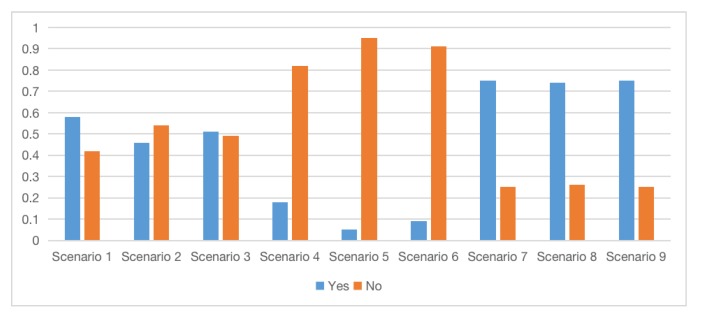
Estimated probabilities of “J. Whether the attack is successful” under different scenarios.

From Figure 5b–d, the attractions of different targets are quite different. The attraction of Target A and B is higher, and the attraction of Target C is very low. At the same time, it can be obtained from Figure 5c that police investigation and security check have an important impact on the police’s defense capabilities. The same conclusion also emerges in the defense methods for controlling the risk of urban dirty bomb attacks. Compared with patrol and surveillance, security inspections are more important and effective [56]. Because Target A lacks “Police investigation” and does not have “security check”, the probability of “High” in node G dropped from 0.98 to 0.13. The combination of different prevention methods has an impact on the overall risk level. For example, when patrol, police investigation, surveillance, and security check are all at high levels, the police have a strong ability to combat and prevent. In Figure 6, the probability of successful attacks is reduced by nearly 40 percentage points compared with only have detection and monitoring measures (0.58–0.18, 0.46–0.05, 0.51–0.09). Compared with the non-preventive measures, the probability of successful terrorist attacks is reduced with nearly 60 percentage points (0.75–0.18, 0.74–0.05, 0.75–0.09). If there are only the two most common measures for patrol and monitoring and deterrence, it still can reduce the probability of successful attacks by at least 15 percentage points. In real life, due to limited defense resources and limited terrorist attack resources, the network can be used to evaluate the most effective and comprehensive prevention measures to reduce the overall level of risk.

**Figure 7:**
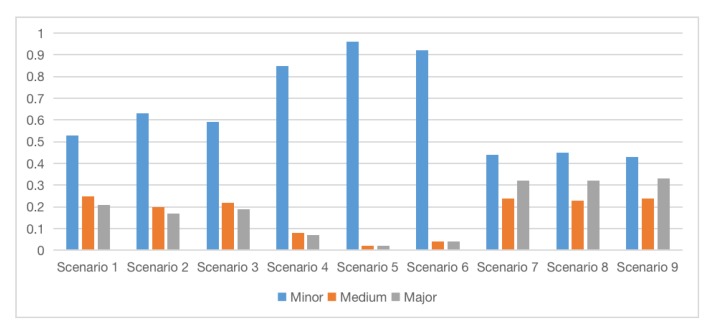
Estimated probabilities of “K. Casualties” under different scenarios.

Figure 6 shows the probability distribution of “J. Whether the attack is successful”. Scenario 5 has the highest probability of failure, reaching 0.95. That’s because scenario 5 indicates that terrorist organisation 2 attacks the government. From Figure 5c, we find the government’s defense and emergency capabilities are strong. At the same time, the threat degree of Organisation 2 is low, so it is understandable to get such result. The probability of failure is low in scenario 7, 8, and 9, where the lowest one is 25%. Since Target C is in an open area, the probability of effective early warning is low, so the attack is more likely to succeed [56]. We can infer that terrorist organisations with different threat levels have a limit impact on the success of the attack, while the police prevention in different locations has a more significant impact. Figure 7 shows the probability distribution of “K. Casualties.” The impact of emergency response capabilities on casualties are more significant than terrorist organisations of different threat levels. One of the reasons is that node I is not directly connected to node K. However, rather than reducing the direct casualties, emergency response is more significant in conducting rescue operations, reducing disasters such as continuous chemical pollution caused by chemical substances, moving casualties from contaminated environment to well-ventilated area to give first aid [1], and reducing the possibility of secondary attacks [40,56]. These measures ultimately reduced casualties. At the same time, among Scenario 1, 2, and 3, the probability of minor casualties in Scenario 2 is the highest, because Organisation 2 has the lowest threat. Finally, we conclude that the probability of an attack failure is negatively related to casualties, which is clearly consistent with common sense.

Then we consider the impact of different chemical weapon types, doses, delivery methods on casualties. We use scenario 3 as an example. Scenario 3 represents Organisation 3 attack the Target A. When we changed the chemical weapon type to neurotoxin, the state “minor” dropped from 0.59 to 0.57, the state “major” increases from 0.19 to 0.24. The lowest casualties are corrosive poisons and uncertain types, with “minor” is 0.59 and “major” is 0.18. This means that neurotoxic agents are more dangerous, while corrosive agents are less dangerous. When the release method is explosive dispersion, the “minor” is 0.57 and the “major” is 0.24, which is the most dangerous of all delivery methods. When the chemical weapon dose is set to the maximum, the “minor” is 0.57 and the “major” is 0.24; when the dose is set to be small, the “minor” is 0.59, and the “major” is 0.2. In the simulation of dirty bomb incidents, it is very important to predict the concentration of radioactive materials in the environment of an emergency [50,57]. This is also important in chemical terrorist attacks. When we set the weapon danger to “high,” the “minor” dropped from 0.59 to 0.49, and the “major” rose from 0.19 to 0.49. It can be seen that the danger level of weapon has a strong impact on casualties. When we set the weapon danger to low, the “minor” rises from 0.59 to 0.68 and the “major” drops from 0.19 to 0.1. We do not repeat other similar analysis.

Threat (or risk) assessment for different scenarios is significant. Olama found that when the likelihood of an attack is low/high, and the consequence of attack is low/high, the threat assessment is low/high as well [42]. So, we can infer from Figure 6 and Figure 7 that the threats of scenario 7, 8, 9 are the highest, while the threats of scenario 4, 5, 6 are the lowest. The assumption is not consistent with the actual situation. Because when setting the node status, we focus on exploring the police’s defense capabilities and the role of emergency response capabilities, so that we take the extreme state of “yes or no”. Scenario 1, 2, 3 are more likely to happen in reality. As Target B is attractive, and the defense has some loopholes, the terrorist is more likely to receive the maximum return. While the attraction of Target C is low, the probability of a terrorist attack is rather low. The situation explains a game of attack resources and defense resources [58,59]. Moreover, Cox [60] pointed out the limitations of several important risk analysis methods based on the formula: “Risk = Threat × Vulnerability × Consequence.” The two-level (or few-level) hierarchical optimisation models appear valuable as practical approaches to antiterrorism risk analysis and protect critical targets.

What’s more, the reference [38] found that under the threat of nuclear or biological weapons terrorist attacks, if the defender wants to protect the target, the priority of the protected target should be Government Buildings, Symbolic Buildings, Urban Populations, Low value target. Such a priority allows the defender to get the highest benefit while minimizing the probability of successful attacks. This finding is consistent with the defensive power setting of three targets. Among the targets, the government’s defensive strength is the strongest; the downtown shopping mall is the second; and the suburban defensive strength is the worst. Moreover, we can see in Figure 6 and Figure 7 that the probability of a successful attack on government and the casualties are the smallest. The probability of a successful attack on Suning square is medium, and the park has the largest probability of a successful attack and casualties.

## 5. Conclusions 

In this study, we use the idea of module construction to make the complex issues simplified. The critical nodes are integrated into a comprehensive Bayesian network to estimate the dangerous level of the chemical weapon, target attraction, weather condition, prevention ability of the police, ability of the emergency response, threat of the terrorist organisation, whether the attack is successful and casualties. In order to adequately estimate the impact of each parent node, we analyse case data, combining expert knowledge to identify key nodes and determine status values. Based on expert knowledge, we construct Bayesian network. The probability distribution of the network is obtained using the EM algorithm and expert experience. We perform different scenario analysis to predict the possible impacts and consequences. Sensitivity analysis is used to identify crucial and influential nodes and their impact on the consequences of an attack. In order to prove the validity and applicability of the proposed method, a case study of Lianyungang City is further carried out. By performing nine different scenarios in the case study, the feasibility and correctness of the model has been verified.

The models and procedures in this article can be used to complete similar terrorist attack modeling and risk analysis in other regions. However, the model structure and probability distribution need to be changed based on regional differences. Furthermore, the risk analysis conclusions from this article can be used by government to effectively allocate defense resources and minimise the risk of chemical terrorist attacks. Because of the graphical nature of Bayesian network, it is easy for public safety officials to understand the interactions between key factors and to add new evidence to infer risks and threats. Users can further improve the performance of the proposed model based on specific weather conditions, more detailed emergencies, and other relevant information.

This article still has some limitations. First, the chemical weapon terrorist attack model and analysis method proposed in this paper lack the knowledge base support, which may affect the practicality of our model. If we can combine the personnel information system and geographic information system or use a knowledge graph that integrates multi-source knowledge, the practicality of the model will be significantly enhanced. Another limitation is the risk assessment of terrorist attacks. This article does not obtain specific attack risk indicators or values. If a precise method is used to calculate the risk value, it can better support decision-making. For future research, this Bayesian network model can be integrated with a knowledge graph or knowledge base to develop a risk assessment system. We will consider using game theory to assess the level of attraction of targets to support decision-making. Another possible research direction is to develop a plan support system [61,62] based on our proposed BN model and conduct a comprehensive analysis of the system’s objectivity, rationality, understandability, and usefulness.

## Figures and Tables

**Table 1:** States of Bayesian Network (BN) nodes.

No.	Nodes	States of Bayesian Nodes
1	Religious background	(1) Cult terrorism; (2) Islamic terrorism; (3) Christian terrorism; (4) Jewish terrorism; (5) Other
2	Region of the perpetrator	(1) Middle East and North Africa; (2) Europe; (3) Americas; (4) South and Southeast Asia; (5) East and Central Asia; (6) Central and North Africa
3	Number of members	(1) Less than 50 people; (2) 50–500 people; (3) 500–5000 people; (4) More than 5000 people
4	Average educational level	(1) High; (2) Medium; (3) Low
5	Technical background	(1) High level; (2) Middle level; (3) Low level
6	Social relations and organisational components	(1) Complex and diverse; (2) Medium; (3) Single
7	Whether they have been reported recently	(1) Yes; (2) No; (3) Unknown
8	Whether they ever launched chemical attack	(1) Yes; (2) No
9	Whether they made a statement or threat	(1) Yes; (2) No
10	Source of chemical weapon	(1) Self-made; (2) Occupied inventory or armory; (3) Steal from elsewhere; (4) Black market
11	Whether they have technical support	(1) Yes; (2) No
12	Whether they have the capabilities of storing and transporting the chemical weapon	(1) Yes; (2) No
13	Whether they have the ability to launch the chemical attack	(1) Yes; (2) No
14	Weapon types	(1) Irritant agent; (2) Erosive agent; (3) Systemic poison; (4) Neurotoxic agent; (5) Asphyxiating agent; (6) Acid and alkali corrosive weapons; (7) Mixed Poison; (8) Unknown
15	Delivery method	(1) Volatile; (2) Water-soluble; (3) Spraying; (4) Explosive dispersion; (5) Send by post; (6) Unknown
16	Chemical dose	(1) Large; (2) Medium; (3) Little; (4) Unknown
17	Population density	(1) >1000/km^2^; (2) 500–1000/km^2^; (3) <500/km^2^
18	Population movement	(1) High; (2) Medium; (3) Low
19	Traffic condition	(1) Good; (2) Bad
20	Location	(1) Residential area; (2) Commercial area; (3) Open space
21	Whether it is a high-value target	(1) Yes; (2) No
22	Wind speed	(1) ≤2 m / s; (2) 2 m / s ~ 4 m / s; (3) > 4 m / s
23	Wind direction	(1) Upwind; (2) Downwind
24	Precipitation	(1) Heavy; (2) Medium; (3) Less; (4) Minimal or dry
25	Patrol	(1) More than 2 times; (2) Less than 2 times
26	Security check	(1) Yes; (2) No
27	Surveillance	(1) 24 h; (2) Non-24 h
28	Police investigation	(1) Yes; (2) No
29	Hospital emergency response	(1) On time; (2) Delay
30	Fire emergency response	(1) On time; (2) Delay
31	Police emergency response	(1) On time; (2) Delay
A	Influence of the terrorist organisation	(1) Large; (2) Medium; (3) Small
B	Activity level of the terrorist organisation	(1) Inactive; (2) Active; (3) Very active
C	Difficulty in obtaining and using the chemical weapon	(1) Low; (2) Medium; (3) High
D	Danger level of the chemical weapon	(1) High; (2) Medium; (3) Low
E	Target attraction	(1) High; (2) Medium; (3) Low
F	Weather condition	(1) Favorable; (2) Unfavorable
G	Prevention ability of the police	(1) High; (2) Medium; (3) Low
H	Ability of the emergency response	(1) High; (2) Medium; (3) Low
I	Threat of the terrorist organisation	(1) Large; (2) Medium; (3) Small
J	Whether the attack is successful	(1) Yes; (2) No
K	Casualties	(1) Minor (0 to 10 deaths or 0 to 50 injuries); (2) Medium (11 to 30 deaths or 50 to 100 injuries); (3) Major (more than 30 deaths or more than 100 injuries)

**Table 2:** Partial probability questionnaires and weighted conditional probabilities of BN nodes.

Nodes			Experts’ Opinion				Calculated Results
Hospital Emergency Response	Fire Emergency Response	Police Emergency Response	m1(1,2,3)	m2(1,2,3)	m3(1,2,3)	m4(1,2,3)	m(1,2,3)
(1) On time	(1) On time	(1) On time	(0.9,0.09,0.01)	(0.9,0.05,0.05)	(0.9,0.08,0.02)	(0.95,0.04,0.01)	(1,0,0)
(1) On time	(1) On time	(2) Delay	(0.4,0.35,0.25)	(0.75,0.15,0.1)	(0.5,0.3,0.2)	(0.8,0.15,0.05)	(0.979,0.019,0.002)
(1) On time	(2) Delay	(1) On time	(0.3,0.3,0.4)	(0.7,0.2,0.1)	(0.4,0.3,0.3)	(0.7,0.2,0.1)	(0.925,0.057,0.019)
(1) On time	(2) Delay	(2) Delay	(0.03,0.17,0.8)	(0.5,0.3,0.2)	(0.1,0.3,0.6)	(0.2,0.5,0.3)	(0.008,0.208,0.784)
(2) Delay	(1) On time	(1) On time	(0.45,0.25,0.3)	(0.6,0.2,0.2)	(0.45,0.3,0.25)	(0.7,0.2,0.1)	(0.950,0.034,0.017)
(2) Delay	(1) On time	(2) Delay	(0.05,0.2,0.75)	(0.4,0.3,0.3)	(0.15,0.3,0.55)	(0.15,0.35,0.5)	(0.007,0.092,0.902)
(2) Delay	(2) Delay	(1) On time	(0.05,0.2,0.75)	(0.3,0.25,0.45)	(0.07,0.3,0.63)	(0.1,0.3,0.6)	(0.001,0.034,0.965)
(2) Delay	(2) Delay	(2) Delay	(0.01,0.09,0.9)	(0.01,0.01,0.98)	(0.01,0.09,0.9)	(0.01,0.04,0.95)	(0,0,1)

**Table 3:** Extreme-Condition Test of the proposed Bayesian Network.

No	Parent Nodes	Extreme Worst	Extreme Best
1	Religious background	Islamic terrorism	Jewish terrorism
2	Region of the perpetrator	Middle East and North Africa	Central and North Africa
3	Number of members	More than 5000 people	Less than 50 people
4	Average educational level	High	Low
5	Technical background	High level	Low level
6	Social relations and organisational components	Complex and diverse	Single
7	Whether they have been reported recently	Yes	No
8	Whether they ever launched chemical attack	Yes	No
9	Whether they made a statement or threat	Yes	No
10	Source of chemical weapon	Self-made	Steal from elsewhere
11	Whether they have technical support	Yes	No
12	Whether they have the capabilities of storing and transporting the chemical weapon	Yes	No
13	Whether they have the ability to launch the chemical attack	Yes	No
14	Weapon types	Irritant agent	Acid and alkali corrosive weapons
15	Delivery method	Explosive dispersion	Spraying
16	Chemical dose	Large	Little
17	Population density	>1000/km^2^	<500/km^2^
18	Population movement	High	Low
19	Traffic condition	Good	Bad
20	Location	Commercial area	Open space
21	Whether it is a high-value target	Yes	No
22	Wind speed	>4 m/s	≤2 m/s
23	Wind direction	Downwind	Upwind
24	Precipitation	Minimal or dry	Many
25	Patrol	Less than 2 times	More than 2 times
26	Security check	No	Yes
27	Surveillance	Non-24 h	24 h
28	Police investigation	No	Yes
29	Hospital emergency response	Delay	On time
30	Fire emergency response	Delay	On time
31	Police emergency response	Delay	On time
G	Prevention ability of the police	High: 0% Medium: 0% Low: 100%	High: 100% Medium: 0% Low: 0%
H	Ability of the emergency response	High: 0% Medium: 0% Low: 100%	High: 100% Medium: 0% Low: 0%
I	Threat of the terrorist organisation	Large: 54% Medium: 23% Small: 23%	Large: 35% Medium: 34% Small: 32%
J	Whether the attack is successful	Yes: 86% No: 14%	Yes: 0% No: 100%
K	Casualties	Minor: 24% Medium: 16% Major: 59%	Minor: 100% Medium: 0% Major: 0%

**Table 4:** Results of four different scenarios.

Parent Nodes	Scenario 1	Scenario 2	Scenario 3	Scenario 4
Whether the attack is successful	Yes: 46% No: 54%	Yes: 46% No: 54%	Yes: 51% No: 49%	Yes: 24% No: 76%
Casualties	Minor: 69% Medium: 27% Major: 4%	Minor: 61% Medium: 16% Major: 23%	Minor: 66% Medium: 29% Major: 5%	Minor: 84% Medium: 14% Major: 2%

**Table 5:** Sensitivity of “Casualties” to patrol, security check, surveillance, police investigation.

Value of Nodes 25–28	Casualties	Patrol	Security Check	Surveillance	Police Investigation
yes	minor	78%	90%	77%	88%
	medium	12%	5%	13%	6%
	major	10%	5%	10%	6%
no	minor	65%	66%	61%	71%
	medium	20%	19%	22%	16%
	major	15%	15%	17%	13%

**Table 6:** Sensitivity of “Whether the attack is successful” to patrol, security check, surveillance, police investigation.

Value of Nodes 25–28	Whether the Attack is Successful	Patrol	Security Check	Surveillance	Police Investigation
yes	yes	28%	13%	30%	16%
	no	72%	87%	70%	84%
no	yes	47%	45%	52%	39%
	no	53%	55%	48%	61%

**Table 7:** Bayesian network node status at three locations.

Parent Nodes	Target A	Target B	Target C
Population density	> 1000/km^2^	500–1000/km^2^	<500/km^2^
Population movement	High	Medium	Low
Traffic condition	Good	Good	Bad
Location	Commercial area	Residential area	Open space
Whether it is a high-value target	Yes	Yes	No
Patrol	More than 2 times	More than 2 times	Less than 2 times
Security check	No	Yes	No
Surveillance	24 h	24 h	Non-24 h
Police investigation	No	Yes	No
Hospital emergency response	On time	Delay	Delay
Fire emergency response	On time	On time	Delay
Police emergency response	On time	On time	Delay

**Table 8:** Bayesian network node status of three terrorist organisations.

Parent Nodes	Organisation 1	Organisation 2	Organisation 3
Religious background	Islamic terrorism	Christian terrorism	Cult terrorism
Region of the perpetrator	Middle East and North Africa	Europe	East and Central Asia
Number of members	More than 5000 people	50–500 people	Less than 50 people
Average educational level	Middle	Low	High
Technical background	Middle level	Low level	High level
Social relations and organisational components	Complex and diverse	Medium	Single
Whether they have been reported recently	Yes	No	No
Whether they ever launched chemical attack	Yes	No	No
Whether they made a statement or threat	Yes	No	No
Source of chemical weapon	Occupied inventory or armory	Steal from elsewhere	Self-made
Whether they have technical support	No	No	Yes
Whether they have the capabilities of storing and transporting the chemical weapon	Yes	No	Yes
Whether they have the ability to launch the chemical attack	Yes	No	No

**Table 9:** Description of nine scenarios.

Scenario	Description	Scenario	Description	Scenario	Description
Scenario 1	Organisation 1 Target A	Scenario 4	Organisation 1 Target B	Scenario 7	Organisation 1 Target C
Scenario 2	Organisation 2 Target A	Scenario 5	Organisation 2 Target B	Scenario 8	Organisation 2 Target C
Scenario 3	Organisation 3 Target A	Scenario 6	Organisation 3 Target B	Scenario 9	Organisation 3 Target C

## Appendix A

1. **Religious background**. This node comes from the religious types of terrorist organisations recorded in the GTD data that have carried out chemical terrorist attacks, which is divided into five categories. The state “unknown” means individuals or unknown organisations launched the attack [38,42].
2. **Region of the perpetrator**. This node refers to the region of the attacker. By reviewing literature and historical cases, we divide the node into eight categories [3,39].
3. **Number of members**. The number of terrorist organisations is an important indicator of their influence. We divide the number of organisations into four categories [39,40].
4. **Average educational level**. The average education level is divided into three categories: high, medium and low. Low means that members are mostly below primary school level, high means members include many college diplomas, doctorates, or researchers. The medium means the members have a high school education level [42].
5. **Technical background**. There are three technical backgrounds, high, medium and low. The technical background is whether there is background knowledge related to chemistry and weapon manufacturing. With this kind of knowledge, the organisation is more dangerous [42].
6. **Social relations and organisational components**. High-impact terrorist organisations generally have complex social relationships. Moreover, it is easier to launch attacks through complex social relations and more difficult to prevent. We manually assign the specific values by looking up information about terrorist organisations in Wikipedia and related news [42].
7. **Whether they have been reported recently**. We look at similar terrorist attacks or attacks of the same organisation in the GTD. If the time interval is within one month, then the organisation has been reported and needs to be vigilant.
8. **Whether they ever launched chemical attack**. We check in the GTD to see if any attacks were carried out using similar means in similar locations, or if a terrorist organisation has repeatedly carried out chemical terrorist attacks.
9. **Whether they made a statement or threat**. Some terrorist organisations will threaten and intimidate before the attack, causing people to panic. Terrorist organisations that make such statements are often more active.
10. **Source of chemical weapon**. We have summarised six ways through the literature, including self-made, obtained from the occupied inventory, and purchased from the black market. We obtain such information from relevant reports and Wikipedia [38,42].
11. **Whether they have technical support**. We look for information on terrorist organisations on the Internet and perform manual evaluations and assignments [38].
12. Whether they have the capabilities of storing and transporting the chemical weapon. We manually assign values based on GTD and Wikipedia data.
13. **Whether they have the ability to launch the chemical attack**. We manually assign values based on GTD and Wikipedia data, such as the ordinary attacks that the terrorist organisation has launched [38].
14. **Weapon types**. By reviewing literature [3,4] and historical cases, we divide weapon types into seven types: (1) Neurotoxic agent, which is a class of agents that can damage the nervous system, mainly includes sarin, soman, and VX. People can cause poisoning through inhalation or skin absorption. Nerve agents are fast-killing, extremely lethal, and deadly. (2) Erosive poison. It is a kind of poison that can make cells and tissues necrotic and ulcerated, mainly including mustard gas and Louis gas. People cause poisoning through inhalation or skin contact. The poisoning effect is usually slow. (3) Systemic toxic agent. It is a class of poisons that can destroy the oxidative function of tissue cells, mainly includes hydrocyanic acid and cyanogen chloride. (4) Disabled poison. It is a poison that can cause mental and motor dysfunction and temporarily deprive personnel of combat effectiveness. People can cause poisoning through inhalation, and the poisoning effect is relatively rapid. (5) Choking poison. It is a type of poison that stimulates the respiratory tract and causes pulmonary oedema and suffocation, mainly includes phosgene. People can cause poisoning through inhalation, and the poisoning effect is slow. (6) Irritating poison. It is a class of poisons that can irritate the eyes, upper respiratory tract and skin, mainly includes Siez (CS), acetophenone, Adam’s gas, people can cause poisoning through inhalation and contact. (7) Corrosive objects, mainly a mixture of acid and alkali, such as nitric acid, sulfuric acid, sodium hydroxide, these poisons play a corrosive role. (8) Mixed poisons refer to a mixture of multiple poisons that cannot be accurately classified [39,42].
15. **Delivery method**. Hu concluded that the impact of the form of emission of nuclear substances on the diffusion distribution is hugely significant [52]. For example, nuclear terrorist attacks are mostly realised by explosions, the discharge time is short, the initial velocity is large, and the diffusion distribution is broader and quicker. Chemical substances have similar characteristics. Chemicals can be released in a variety of ways, including volatilisation, water-solubility, spraying, and explosive dispersion [38].
16. **Chemical dose**. Even small amounts of neurotoxins are still very dangerous, corrosive toxins are often more abundant. Therefore, we added the node and divide it into four categories: large, medium, small and unknown. The dose of chemical weapons directly affects the level of bomb explosions and casualties [56].
17. **Population density**. According to the geographic information system (GIS) of the case studied, we divided population density into three levels, namely “over 1000 people/km^2^”, “500 to 1000 people/km^2^” and “less than 500 people/km^2^”. Population density directly affects the number of casualties caused by bomb explosions. As in the areas with high population density, the risk of serious casualties will increase [42].
18. **Population movement** There are three types of population movement: high, medium and low. For example, the population movement in subway stations and shopping malls is high, while the level of government is medium, the level of some remote residential areas or parks is low.
19. **Traffic condition**. Traffic condition refer to whether it is conducive to evacuation, rescue, rapid response. In transportation hubs such as railway stations, the traffic conditions are better, but in the suburbs and mountain areas, the traffic conditions are poor.
20. **Location**. In the case study, chemical terrorist attacks may have occurred in three different types, namely “commercial area”, “residential area” and “open space”. Obviously, different places have different requirements for defensive forces [38,42,56].
21. **Whether it is a high-value target**. High-value locations includes: urban lifeline systems such as transportation, energy, communications, water supply and drainage; densely populated places, such as hotels, restaurants, shopping malls, bazaars, stadiums, halls, and public entertainment venues and other public gathering places; important buildings and places: such as government office buildings, stadiums, halls; the important security goal, including humanities and historical sites, the army, and public security organs [38,39,42].
22. **Wind speed**. We have borrowed from the simulation of the risk evolution of the “dirty bomb” terrorist attack [50,52,57]. The diffusion of chemical substances is similar to the dirty bomb, so we have added wind speed and direction to the weather condition. We divide wind speed into three states according to the actual situation of the case they study, namely “less than 2 m/s”, “2 m/s to 4 m/s” and “more than 4 m/s”.
23. **Wind direction**. The wind direction during a chemical terrorist attack can be either upwind or downwind. Downwind means that the wind direction is conducive to the spread of chemical substances, while upwind will hinder and reduce the impact of incidents [50,52,56,57].
24. **Precipitation**. Hu pointed out that wind and precipitation have a significant effect on the atmospheric diffusion of nuclear matter [52]. Chemicals have similar atmospheric diffusion characteristics. According to the precipitation of Lianyungang, there are four levels of precipitation: (1) heavy; (2) medium; (3) less; (4) very little or dry.
25. **Patrol**. Police usually walk around to check the highly attractive locations which is more dangerous. In terms of patrol frequency of our case, there are two states:”> two times” and “<two times” [56]
26. **Security check**. Security check are designed to detect contraband and dangerous people by inspecting people or vehicles in an area. We classify security check into yes and no [56].
27. **Surveillance**. Surveillance means monitoring a specific area and sending images or video data to the police to assess the safety of the public. We divide monitoring into two states: “24 h coverage” and “non-24 h coverage” [56].
28. **Police investigation**. We use “yes” and “no” to indicate whether chemical weapon attacks can be detected in advance [56].
29. **Hospital emergency response**. The node means that after a chemical terrorist attack, emergency response measures such as rapid hospital rescue can be divided into “on time” or “delayed.”
30. **Fire emergency response**. Fire emergency response means that after a chemical terrorist attack
31. **Police emergency response**. Public security emergency response refers to quickly control the order of an incident and organise evacuation after a chemical terrorist attack.

- **A. Influence of the terrorist organisation**. Terrorist organisations with different influences have different threats. For example, ISIS has a greater influence, and its threat is also greater [38].
- **B. Activity level of the terrorist organisation**. The active level of a terrorist organisation has a strong relationship with his current threat level. The organisations that have repeatedly launched terrorist attacks recently are more prone to the vigilance of the defense than the terrorist organisations that have not heard of it for a long time.
- **C. Difficulty in obtaining and using the chemical weapon**. The difficulty of acquiring weapons determines whether terrorist organisations can launch multiple chemical weapons attacks and reflects the degree of threat [38].
- **D. Danger level of the chemical weapon**. Different levels of chemical weapons cause different casualties. The degree of danger is influenced by weapon type, dose, and delivery method [38].
- **E. Target attraction**. Different objectives have different appeal to terrorists. We divide target attraction into high, medium and low. The different targets have an impact on the success of the attack [42]. The degree of target attraction is influenced by population density, population movement, traffic condition, location, whether it is a high-value target.
- **F. Weather condition**. Different climatic conditions have an impact on the success of the attack [52]. Weather condition is affected by wind speed, wind direction and precipitation
- **G. Prevention ability of the police**. High vulnerability objectives can be easily attacked. Prevention ability of the police is affected by patrol, security check, surveillance and police investigation. Different preventive capabilities have an impact on the success of the attack [38].
- **H. Ability of the emergency response**. Emergency response capabilities are mainly composed of public security, hospitals, and fire forces. With rapid response and timely diagnosis, a variety of chemical agents can be treated, especially neurotoxic drug poisoning [1,56].
- **I. Threat of the terrorist organisation**. The threat degree is mainly affected by the influence, the active degree, and the difficulty of acquiring weapons of the terrorist organisations. The degree of threat of different terrorist organisations has an impact on the success of the attack [38,42].
- **J. Whether the attack is successful**. We divide the states of the node into “yes” and “no”. The node reflects the likelihood of a successful attack [39,40,42].
- **K. Casualties**. Casualties are essential indicators for assessing the consequences of a chemical weapons attack. We have summarised three types of casualties in chemical terrorist attacks based on the “Safety Production Incident Report”, “Chinese Investigation and Handling Rules”: “less than ten people died or less than 50 people were injured”, “11 to 30 people died or 51 To 100 injuries “and” more than 30 dead or more than 100 injured” [38,39,42,56].
